# Supplementary material for: Essential Role of Multi-Omics Approaches in the Study of Retinal Vascular Diseases
Source: Cells. 2022 Dec 26;12(1):103. doi: 10.3390/cells12010103 (PMC9818611; doi:10.3390/cells12010103)
Supplement: Supplementary file 1 [file cells-12-00103-s001.zip › cells-2109280-supplementary.pdf]

Table S1. Omics biomarkers for DR

Reference	Accession	Source	Type	Organism	Specimen	Biomarkers/Findings	Purpose
Skol(2020)[1]	GSE146615	GEO	genomics and transcriptomics	human sapiens	lymphoblastoid cell lines	FLCN	Diagnosis and staging
He(2022)[2]	GSE193974	GEO	transcriptomics	human sapiens	blood	hsa_circ_0095008 and hsa_circ_0001883	Diagnosis
Li(2021)[3]	GSE178721	GEO	transcriptomics	human sapiens	serum	circFndc3b	Diagnosis and staging
Han(2020)[4]	GSE140842	GEO	genomics	human sapiens	blood	5-hydroxymethylcytosines	Diagnosis and prognosis
Smit-McBride (2020)[5]	GSE140959	GEO	transcriptomics	human sapiens	aqueous, vitreous, and serum	let-7b, miR-320b, miR-762 and miR-4488	Diagnosis and staging
Chee(2016)[6]	PXD003723	Pride	proteomics	human sapiens	salivary	Alpha-1-antitrypsin (SERPINA1)	Diagnosis and staging
Amorim(2022)[7]	PXD033101	Pride	proteomics	human sapiens	tear	IL-2/-5/-18/-13, TNF, MMP-2/-3/-9	Diagnosis and staging
Mammadzada(2019)[8]	S-EPMC6947310	Biostudies	transcriptomics	human sapiens	vitreous	miRNA-20a and -93	Prognosis
Sundstrom(2018)[9]	PXD008989	Pride	proteomics	human sapiens	retina	Dopamine (DA)	Screening or early stage DR
Yang(2022)[10]	PXD030660	ProteomeXchange	Metabolomics and Proteomics	human sapiens	serum	FIBA and 1-MH	Diagnosis and staging
Guo(2022)[11]	MTBLS4250	MetaboLights	Metabolomics	human sapiens	serum	Thiamine metabolism and tryptophan metabolism	Screening or diagnosis

Chen(2016)[12]	GSE76171	GEO	Epigenomics	human sapiens	blood	DNA-methylation at key DMLs	Screening or diagnosis

Table S2 Omics biomarkers for AMD

Article	Accession	Source	Data Type	Originate	Tissue	Biomarkers	Purpose
Ratnapriya (2019)[13]	GSE115828	GEO	Genomics<br>Transcriptomics	Homo Sapiens	retina	GWAS&TWAS：B3GALTL, RDH5/CD63, SLC16A8 (rs5756908), ACAD10 (rs7398705), TMEM/VTN (rs241777), and APOE (rs157580), TWAS：RLBP1, HIC1 and PARP12	Etiology<br>Dignosis
Park(2009)[14]	phs000182.v2.p1	EGA	Genomics	Homo Sapiens	blood	SERPING1	Diagnosis, prognosis, pathology
Wei(2012)[15]	GSE28033	GEO	Transcriptomics<br>Epigenomic	Homo Sapiens	blood	IL17RC	Diagnosis,staging
ElShelmani(2021)[16]	S-EPMC8625913	Biostudies	Transcriptomics	Homo Sapiens	serum	hsa-let-7a-5p, hsa-let-7d-5p, hsa-miR-23a-3p, hsa-miR-301a-3p	Screening
ElShelmani(2020	S-EPMC739	Biostudies	Transcripto	Homo	serum	miR-126, miR-410, and miR-19a	Diagnosis, staging,

)[17]	6178		mics	Sapiens			prognosis
Ertekin(2014）[18]	S-EPMC4113960	Biostudies	Transcriptomics	Homo Sapiens	plasma	miR-17-5p, miR-20a-5p, miR-24-3p, miR-106a-5p, and miR-223-3p	Diagnosis
Mantel(2020)[19]	S-EPMC7802772	Biostudies	Proteomics	Homo Sapiens	Aqueous humor	sVCAM-1, IL-6, IL-12p40, PAI-1, and HGF	prognosis and treatment response
Coronado(2021)[20]	MSV000083731	MassIVE	Proteomics	Homo Sapiens	Aqueous humor	IGHM, KLKB1, KDR, VEGFR-1	prognosis and treatment response
Chen(2017)[21]	MTBLS417	MetaboLights	Metabolomics	Homo Sapiens	serum	docosahexaenoic acid (DHA)	Diagnosis
Schori(2018)[22]	PXD008354	Pride	Proteomics	Homo Sapiens	vitreous	Cholinesterase, ribonuclease, serine carboxypeptidase	Diagnosis,staging

References

1. Skol, A.D.; Jung, S.C.; Sokovic, A.M.; Chen, S.; Fazal, S.; Sosina, O.; Borkar, P.P.; Lin, A.; Sverdlov, M.; Cao, D.; et al. Integration of genomics and transcriptomics predicts diabetic retinopathy susceptibility genes. eLife **2020**, 9, e59980.
2. He, H.; Zhang, J.; Gong, W.; Liu, M.; Liu, H.; Li, X.; Wu, Y.; Lu, Q. Involvement of CircRNA Expression Profile in Diabetic Retinopathy and Its Potential Diagnostic Value. Front. Genet. **2022**, 13, 833573.
3. Li X, Wang J, Qian H, et al. Serum Exosomal Circular RNA Expression Profile and Regulative Role in Proliferative Diabetic Retinopathy. Front Genet. **2021**. 12: 719312.
4. Han, L.; Chen, C.; Lu, X.; Song, Y.; Zhang, Z.; Zeng, C.; Chiu, R.; Li, L.; Xu, M.; He, C.; et al. Alterations of 5-hydroxymethylcytosines in circulating cell-free DNA reflect retinopathy in type 2 diabetes. Genomics **2021**, 113 Pt 1, 79–87.
5. Smit-McBride, Z.; Nguyen, A.T.; Yu, A.K.; Modjtahedi, S.P.; Hunter, A.A.; Rashid, S.; Moisseiev, E.; Morse, L.S. Unique molecular signatures of microRNAs in ocular fluids and plasma in diabetic retinopathy. PLoS ONE **2020**, 15, e0235541.
6. Chee, C.S.; Chang, K.M.; Loke, M.F.; Angela Loo, V.P.; Subrayan, V. Association of potential salivary biomarkers with diabetic retinopathy and its severity in type-2 diabetes mellitus: A proteomic analysis by mass spectrometry. PeerJ **2016**, 4, e2022.
7. Amorim, M.; Martins, B.; Caramelo, F.; Gonçalves, C.; Trindade, G.; Simão, J.; Barreto, P.; Marques, I.; Leal, E.C.; Carvalho, E.; et al. Putative Biomarkers in Tears for Diabetic Retinopathy Diagnosis. Front. Med. (Lausanne) **2022**, 9, 873483.

8. Mammadzada, P.; Bayle, J.; Gudmundsson, J.; Kvanta, A.; André, H. Identification of Diagnostic and Prognostic microRNAs for Recurrent Vitreous Hemorrhage in Patients with Proliferative Diabetic Retinopathy. J. Clin. Med. **2019**, 8, 2217.
9. Sundstrom, J.M.; Hernández, C.; Weber, S.R.; Zhao, Y.; Dunklebarger, M.; Tiberti, N.; Laremore, T.; Simó-Servat, O.; Garcia-Ramirez, M.; Barber, A.J.; et al. Proteomic Analysis of Early Diabetic Retinopathy Reveals Mediators of Neurodegenerative Brain Diseases. Investig. Ophthalmol. Vis. Sci. **2018**, 59, 2264–2274.
10. Yang, J.; Liu, D.; Liu, Z. Integration of Metabolomics and Proteomics in Exploring the Endothelial Dysfunction Mechanism Induced by Serum Exosomes From Diabetic Retinopathy and Diabetic Nephropathy Patients. Front. Endocrinol. (Lausanne) **2022**, 13, 830466.
11. Guo, C.; Jiang, D.; Xu, Y.; Peng, F.; Zhao, S.; Li, H.; Jin, D.; Xu, X.; Xia, Z.; Che, M.; et al. High-Coverage Serum Metabolomics Reveals Metabolic Pathway Dysregulation in Diabetic Retinopathy: A Propensity Score-Matched Study. Front. Mol. Biosci. **2022**, 9, 822647.
12. Chen, Z.; Miao, F.; Paterson, A.D.; Lachin, J.M.; Zhang, L.; Schones, D.E.; Wu, X.; Wang, J.; Tompkins, J.D.; Genuth, S.; et al. Epigenomic profiling reveals an association between persistence of DNA methylation and metabolic memory in the DCCT/EDIC type 1 diabetes cohort. Proc. Natl. Acad. Sci. USA **2016**, 113, E3002–E3011.
13. Ratnapriya, R.; Sosina, O.A.; Starostik, M.; Kwicklis, M.; Kapphahn, R.J.; Fritsche, L.G.; Walton, A.; Arvanitis, M.; Gieser, L.; Pietraszkiewicz, A.; et al. Retinal transcriptome and eQTL analyses identify genes associated with age-related macular degeneration. Nat. Genet. **2019**, 51, 606–610.
14. Park, K.H.; Ryu, E.; Tosakulwong, N.; Wu, Y.; Edwards, A.O. Common variation in the SERPING1 gene is not associated with age-related macular degeneration in two independent groups of subjects. Mol. Vis. **2009**, 15, 200–207.
15. Wei, L.; Liu, B.; Tuo, J.; Shen, D.; Chen, P.; Li, Z.; Liu, X.; Ni, J.; Dagur, P.; Sen, H.N.; et al. Hypomethylation of the IL17RC promoter associates with age-related macular degeneration. Cell Rep. **2012**, 2, 1151–1158.
16. ElShelmani, H.; Brennan, I.; Kelly, D.J.; Keegan, D. Differential Circulating MicroRNA Expression in Age-Related Macular Degeneration. Int. J. Mol. Sci. **2021**, 22, 12321.
17. ElShelmani, H.; Wride, M.A.; Saad, T.; Rani, S.; Kelly, D.J.; Keegan, D. Identification of Novel Serum MicroRNAs in Age-Related Macular Degeneration. Transl. Vis. Sci. Technol. **2020**, 9, 28.
18. Ertekin, S.; Yıldırım, O.; Dinç, E.; Ayaz, L.; Fidancı, S.B.; Tamer, L. Evaluation of circulating miRNAs in wet age-related macular degeneration. Mol. Vis. **2014**, 20, 1057–1066.
19. Mantel, I.; Borgo, A.; Guidotti, J.; Forestier, E.; Kirsch, O.; Derradji, Y.; Waridel, P.; Burdet, F.; Mehl, F.; Schweizer, C.; et al. Molecular Biomarkers of Neovascular Age-Related Macular Degeneration With Incomplete Response to Anti-Vascular Endothelial Growth Factor Treatment. Front. Pharmacol. **2020**, 11, 594087.
20. Coronado, B.N.L.; da Cunha, F.B.S.; de Oliveira, R.M.; Nóbrega, O.D.T.; Ricart, C.A.O.; Fontes, W.; de Sousa, M.V.; de Ávila, M.P.; Martins, A.M.A. Novel Possible Protein Targets in Neovascular Age-Related Macular Degeneration: A Pilot Study Experiment. Front. Med. (Lausanne) **2021**, 8, 692272.
21. Chen, G.; Walmsley, S.; Cheung, G.C.M.; Chen, L.; Cheng, C.-Y.; Beuerman, R.W.; Wong, T.Y.; Zhou, L.; Choi, H. Customized Consensus Spectral Library Building for Untargeted Quantitative Metabolomics Analysis with Data Independent Acquisition Mass Spectrometry and MetaboDIA Workflow. Anal. Chem. **2017**, 89, 4897–4906.
22. Schori, C.; Trachsel, C.; Grossmann, J.; Zygoula, I.; Barthelmes, D.; Grimm, C. The Proteomic Landscape in the Vitreous of Patients With Age-Related and Diabetic Retinal Disease. Investig. Ophthalmol. Vis. Sci. **2018**, 59, AMD31–AMD40.
